# Current-clamp experiments on primary hippocampal neurons shed light on the role of L-type voltage-gated calcium channels in depolarization shifts

**DOI:** 10.1186/1471-2210-11-S2-A28

**Published:** 2011-09-05

**Authors:** Helmut Kubista, Lena Rubi, Petra Geier, Michael Lagler, Stefan Boehm

**Affiliations:** 1 Department of Neurophysiology and Neuropharmacology, Center for Physiology and Pharmacology, Medical University of Vienna, 1090 Vienna, Austria

## Background

Paroxysmal depolarization shifts are the cellular representations of interictal spikes. Interictal spikes (IIS) have a long history in the diagnosis of epilepsy. Previously, it was thought that IIS are largely asymptomatic, but there is growing evidence that beside being involved in epileptogenesis, they are also involved in the pathogenesis of various other neurological diseases. However, the pathomechanisms leading to the formation of IIS and how IIS may lead to functional neuronal impairment are poorly understood. In a previous study [1] we showed that L-type voltage-gated calcium channels (LTCCs) are capable of augmenting brief neuronal depolarizations. Hence, we were interested in the question whether LTCCs contribute to neuronal depolarization shifts (DS).

## Methods

1 mM caffeine was applied as an epileptogenic agent, and LTCC activity was modulated by Bay K8644 (BayK) and isradipine, respectively.

## Results

In contrast to earlier studies on hippocampal slices [2], caffeine alone failed to induce DS in all but one out of 12 neurons tested. However, when BayK (3 µM) was co-administered, DS were readily evoked in about 60% of 21 neurons tested. DS were abolished by subsequent addition of isradipine (3 µM). Since oxidative stress has been shown to augment LTCC currents, we tested hydrogen peroxide in our DS assay, again employing caffeine co-administration. Within 5 min of exposure to 3 mM H_2_O_2_, DS appeared in a subpopulation of neurons. Testing both BayK and H_2_O_2_ on the same neurons we found that H_2_O_2_ evoked DS only in those neurons where BayK showed a clear DS-inducing effect. In those cells, where BayK failed to cause DS, H_2_O_2_ also had no effect.

## Conclusions

Our data suggest that potentiation of LTCCs promotes the formation of depolarization shifts. Oxidative stress appears to be a pathogenic initiator of DS, and this activity may require LTCCs. Since LTCC augmentation is being considered as a pathological mechanism in neurodegenerative diseases, our data point to the possibility that the initiation of IIS may be a precipitating factor and that LTCC inhibition may provide a means to counteract neuropathogenic mechanisms.

## References

[B1] Geier P Lagler M Boehm S Kubista H Dynamic interplay of excitatory and inhibitory coupling modes of neuronal L-type calcium channels Am J Physiol Cell Physiol 2011 300 C937 C949 10.1152/ajpcell.00219.2010 21228322 PMC3074625

[B2] Moraidis I Bingmann D Lehmenkühler A Speckmann EJ Caffeine-induced epileptic discharges in CA3 neurons of hippocampal slices of the guinea pig Neurosci Lett 1991 129 51 54 10.1016/0304-3940(91)90718-9 1922970

